# Impact of the first year of the “This girl can” physical activity and sport mass media campaign in Australia

**DOI:** 10.1186/s12889-023-15091-2

**Published:** 2023-02-15

**Authors:** Adrian Bauman, Nicola McNeil, Matthew Nicholson, Paul O’Halloran, Emma Seal, Erica Randle, Arthur Stukas

**Affiliations:** 1 Centre for Sport and Social Impact, La Trobe University, Melbourne, VIC Australia; 2 Sydney School of Public Health and Charles Perkins Centre, University of Sydney, 2006 Sydney, NSW Australia; 3 Centre for Sport and Social Impact, Monash University Malaysia, La Trobe University, Kuala Lumpur, Melbourne, VIC Australia; 4 Social and Global Studies Centre, RMIT University, Melbourne, VIC Australia

**Keywords:** Mass media campaign, Physical activity, Sport, Women (gender)

## Abstract

**Introduction:**

Addressing gender inequalities in physical activity is an important public health goal. A major campaign, ‘This Girl Can’ (TGC) was conducted by Sport England from 2015, and TGC was licenced in 2018 by VicHealth in Australia for development and use in a 3-year mass media campaign. The campaign was adapted through formative testing to Australian conditions and implemented within the state of Victoria. The purpose of this evaluation was to assess the initial population impact of the first wave of the TGC-Victoria.

**Methods:**

We assessed campaign impact using serial population surveys, with the target population being women living in Victoria who were not meeting the current physical activity guidelines. Two surveys were carried out before the campaign (October 2017 and March 2018), and the post-campaign survey immediately following the first wave of TGC-Victoria mass media (May 2018). Analyses were primarily on the cohort sample of 818 low-active women followed across all three surveys. We measured campaign effects using campaign awareness and recall, and self-report measures of physical activity behaviour and perceptions of being judged. Changes in perceptions of being judged and in reported physical activity were assessed in relation to campaign awareness over time.

**Results:**

Overall, TGC-Victoria campaign recall increased from 11.2% pre-campaign to 31.9% post-campaign, with campaign awareness more likely among younger and more educated women. There was a slight increase of 0.19 days in weekly physical activity following the campaign. Feeling that being judged was a barrier to physical activity declined at follow up, as did the single item perceptions of feeling judged (P < 0.01). Feeling embarrassed decreased, and self-determination increased, but exercise relevance, theory of planned behaviour and self-efficacy scores did not change.

**Conclusions:**

The initial wave of the TGC-Victoria mass media campaign showed reasonably high levels of community awareness and encouraging decreases in women feeling judged whilst being active, but these did not yet translate into overall physical activity gains. Further waves of the TGC-V campaign are in progress to reinforce these changes and further influence the perception of being judged among low-active Victorian women.

**Supplementary Information:**

The online version contains supplementary material available at 10.1186/s12889-023-15091-2.

## Introduction

Promoting physical activity is an important part of noncommunicable disease prevention and requires integrated and systems wide strategies [1]. Several decades of representative population surveys across many countries have identified that women generally report less physical activity than men and report more structural and life-focused barriers to being active yet have higher rates of intending to be physically active [2–4]. The same pattern is true for adolescent girls and young women [4], and population changes over time in physical activity appear smaller in girls than boys [5]. The differences overall are quite marked, with the absolute prevalence of not meeting physical activity guidelines for women around 8% higher than men, as determined in a study of 168 countries [3]. There is also evidence of substantial gender disparity in the prevalence of sport participation, especially at elite levels [6].

Recent strategies have focused on women in sport and promoting participation in physical activity [7–9]. One of the initial recommended strategies is the use of mass media campaigns and community-wide strategies to change attitudes and social norms towards participation, particularly among women [1].

Towards this goal, the comprehensive “This Girl Can” (TGC) campaign was launched by Sport England in 2015, targeting women of all ages. TGC aimed to change women’s attitudes to feeling embarrassed or judged while participating in sport or exercise, with a population target of increasing the proportion of women participating in physical activity or sport at least once a week [10]. It received substantial support from Sport England in the form of paid mainstream media advertisements, publicity, community events, and support from regional and national sport organisations. TGC earned substantial media coverage and recognition, not only for its physical activity promotion but also for its attempt to change gender stereotypes and community values around activity and sport [10, 11]. The substantial extent of the mainstream media coverage of TGC is shown in Supplementary material Figure S1, peaking at 113 media reports around the TGC-V launch in 2018.

Amongst previous physical activity campaigns, few targeted adolescent girls or reported results that were gender specific; exceptions were a Montreal campaign for adolescent girls [12, 13], a national Australian campaign [14], and another Canadian campaign promoting active travel [15]. For middle-aged women, gender specific effects were reported in a Hawaiian campaign for middle-aged adults [16] and a local community campaign in South Carolina [17]. A few other campaigns reported effects separately for women [18, 19], but most campaigns did not target gender-specific changes in attitudes to physical activity or sport.

Formative message testing has indicated that the message frame and content should be different for women compared to messages targeting all adults [11–13]. Messages for women require different branding of physical activity [13] and should address different attitudes and barriers to inactivity [14]. Although women may be more likely to see or hear mass media campaign messages [20], the subsequent stages of campaign execution should be developed specifically for women [21].

Based on the interest generated by the TGC campaign, the state Health Promotion Foundation in Victoria, Australia (known as VicHealth) licensed it for use in the state of Victoria, Australia. VicHealth explored the meanings of physical activity and sport amongst Victorian women and developed a program logic model for the Victorian campaign. Qualitative formative research examined the perceptions of feeling judged among Victorian women, and identified how women perceived their bodies and social pressures from others led to feelings of being judged and ashamed, and that was a contributor to reducing women’s engagement in physical activity [22]. Different messages were tested and adapted from the English campaign to the multicultural Australian context in Victoria [22]. The campaign aimed to change attitudes to physical activity and sport and increase participation amongst Victorian women, particularly targeting those who were not meeting current physical activity guidelines [23]. Similar to the Sport England TGC campaign, messages were designed for women of all ages, sizes and shapes, with themes focusing on increasing confidence and reducing fears of being judged for being physically active.

The formative research led to the “This Girl Can – Victoria” (TGC-V) campaign, which was launched in March 2018 for an initial three-year period. The campaign comprised paid mass media, a strong digital and website presence, community events and community groups, profiling community supporters and local champions. In addition,, collaboration with elite sport organisations, such as Australian Rules (women) football teams and local sport and recreation agencies (https://www.vichealth.vic.gov.au/programs-and-projects/this-girl-can-vic#). The campaign was overseen by an advisory group that included community and government stakeholders, which provided guidance on the implementation of strategies to target all Victorian women (i.e., including disadvantaged or minority subpopulations).

The aim of this paper is to report on the short-term effects of the TGC-V campaign, comparing two pre-campaign baseline surveys, and then a comparison with the post-campaign survey following wave 1 of the campaign. The primary outcomes were assessed in a cohort of Victorian women and consisted of changes in awareness of the TGC-V campaign, changes in perceptions of feeling judged, and changes in reported weekly physical activity. The primary target group were low active or inactive women in Victoria, Australia, defined as those not meeting recommended physical activity levels for health. Secondary outcomes included measures of perceived relevance of exercise, theoretical measures around attitudes and efficacy towards being active, barriers to exercise and feeling embarrassed about exercise.

## Methods

Process and impact evaluation were used to assess campaign reach and whether it met its objectives. Process evaluation comprised monitoring of community events, campaign supporters and ambassadors, social media usage, paid media delivered, and unpaid public relations. In the first year, the dose of purchased media was delivered through 15 to 60 s media spots, with a total of 700 Target Audience Ratings points (TARPs) in urban areas, and 860 TARPs in rural regions. Impact evaluation was based on population surveys of women aged 18 to 65 from across Victoria, with a defined cohort of 1032 women followed through two sequential pre-campaign surveys (18 October to 3rd November 2017, and 2nd March to 20th March 2018) and a survey following the first wave of TGC-V mass media (7th May to 4th June 2018). Additional analyses on the independent samples (different groups of women at each time point) are shown as Supplementary material.

The data were collected by an accredited online market research panel (TEG Insights). Invitations to complete the survey were sent to a random sample of panel participants who were eligible (female, aged 18–65 and living in the state of Victoria). Quotas were derived by age group and regional/urban residents to match the Australian Bureau of Statistics data for women in Victoria.

### Measures used

The primary self-report outcomes asked of Victorian women were about their physical activity participation and questions about their personal sense of embarrassment and being judged during physical activity, derived from the Sport England TGC campaign evaluation [24]. Physical activity measures included the single item measure in the past week [25, 26] as well as questions allowing stage of change for physical activity to be calculated. Those reporting 5 or more days of activity were likely to be physically active, and those reporting 0–4 days considered ‘low active’ [27]. The stage of change algorithm is based on five categories derived from a readiness to engage in physical activity measure [28].

Being judged was assessed in three ways, all rated on scales of 0 to 10: (i) perceptions of being judged as a barrier to activity; (ii) worry about being judged whilst exercising; and (iii) a single ‘feeling judged’ item. The items on ‘being judged as a barrier’ were reverse coded, so that for all judgement questions, a lower score indicated less worry or embarrassment. Questions are provided in Supplementary Material #1.

Additional questions were asked about perceived relevance of exercise, motivational readiness to change, self-efficacy, theory of planned behaviour and self-determination theory, with items adapted from existing measures to assess attitudes, subjective norms and needs [29–31]. Questions were also asked about recall of generic and campaign specific messages, which also included prompted TGC-specific recall questions.

Summary scores were constructed from groups of individual items in each of the areas above. These scores showed very good to excellent internal consistency with the number of items and Cronbach’s alpha values as follows in the cohort sample at baseline: ‘exercise relevant to me’ scale (8 items, α = 0.77); theory of planned behaviour questions (6 items, α = 0.92); self-determination (4 items, α = 0.85); embarrassed about exercise (7 items, α = 0.89); judgement barriers (3 items, α = 0.91); and worry about being judged (3 items, α = 0.81). Generic campaign awareness was assessed by responses to the question “in the past month have you seen any advertising (such as videos, TV ads, Facebook, Instagram or Twitter posts, website ads, or ads at or on public transport) about exercise and playing sport in relation to women and girls”. Prompted recall was whether they recalled the specific “this girl can” message or logo.

Demographic data comprised age (categorised as 18–34, 35–49 and 50 + years), educational attainment (tertiary education or other), country of birth (COB; Australia/other) and language spoken at home English/other), Indigenous status (yes/no), occupation and income, urban/rural residence and self-reported presence of a disability which limited movement (yes/no).

Statistical methods, using SPSS version 28 (IBM Corp. Released 2021. IBM SPSS Statistics for Windows, 28.0. Armonk, NY: IBM Corp), comprised comparing means across the baseline and follow up time points, using generalised linear models with Bonferroni adjustment for post hoc contrasts, and logistic regression modelling of factors associated with positive changes in physical activity and in feeling judged. Significance is reported at p < 0.05 and p < 0.01 levels. The primary research questions were to assess the impact of the initial TGC-V campaign effects on awareness, feeling judged, and physical activity measures using the target group, the cohort of 818 women who were inactive/low active at baseline.

## Results

The baseline characteristics of survey responders in the cohort, low active cohort and the independent samples are shown in Table 1. The cohort (n = 1032) was similar in age distribution, education, children, occupation, income, Indigenous status, residential location and self-reported disability to the subset of low-active women (n = 818) used in the primary analysis. These cohort characteristics were similar to the independent samples for all variables except for the age category, where the cohort sample was younger. Proportions meeting the five times a week 30 min physical activity level were also consistent.

The data in Table 2 show changes in physical activity, feeling judged, and other attitude measures. Reported physical activity days increased from the baseline to the second pre-campaign measure and were still greater than baseline at follow up. No change was noted in the proportion exercising in the previous week (54.8%, 55.6% and 56.1% respectively), or in the proportion in the action/maintenance stages of change (53.9%, 56.4% and 52.3% post campaign). Although the core data for this analysis is the 818 people who were low active at the first baseline, changes in physical activity levels can be assessed across the cohort. Of those low active at baseline, 69 people increased to meet the guideline level at post-campaign, and 86 relapsed from meeting the guideline at baseline to not meeting the guideline at follow-up.

All measures of being judged improved at the post campaign measure, with reductions in feeling judged as a barrier and the single item ‘feeling judged’ both significantly lower following the campaign. The ‘feeling embarrassed’ score also declined significantly following the campaign. (Table 2). No changes were evident in perceived exercise relevance, theory of planned behaviour, self-determination or physical activity self-efficacy measures.

Campaign awareness was assessed at all timepoints among Victorian low-active women. Recall of any generic message about physical activity and sport for women or girls was similar at both baselines and follow up (respectively 24.6%, 25.1% and 28.9%). Specific *TGC-V recall* was reported by 11.2% at baseline1, 13.2% at baseline2, and 31.9% at post campaign, indicating a significant increase in prompted awareness of the campaign by name (p < 0.01). All women were then shown the TGC messages as part of the online survey, and 71.9% reported the message was easy to understand, and 26.0% said they wanted to find out more. Most felt the message encouraged women to be active (77.9%) and 86.2% thought the message was effective in overcoming the fear of being judged.

**Fig. 1:**
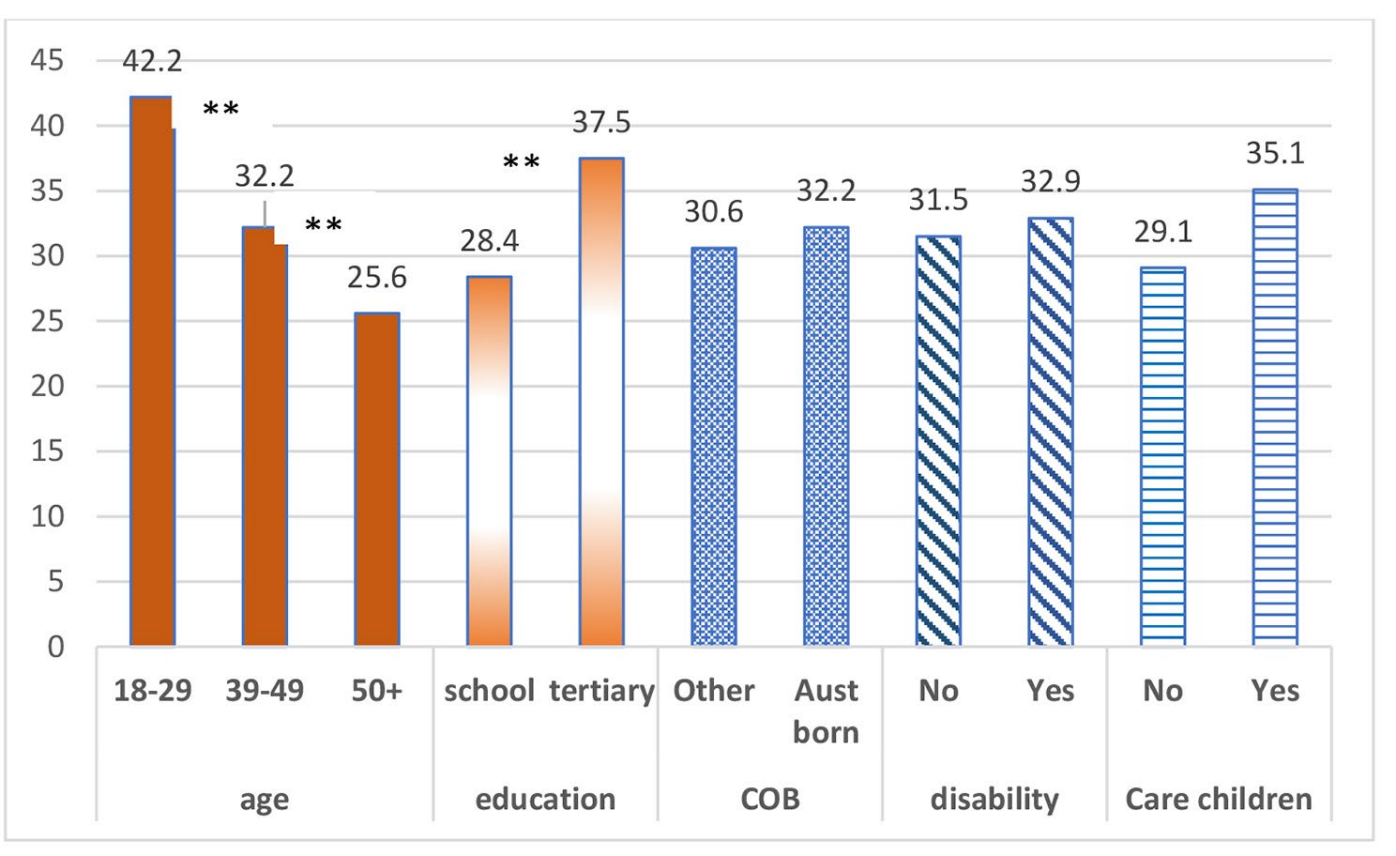
Proportion of initially low active women who reported awareness of the This Girl Can campaign logo, advertisement or both by age, education, country of birth (COB), disability status and whether they care for children (%, among low/inactive, n = 818). (Legend: ** significant differences (P < 0.01)

There were 31.9% of participants who reported recalling the campaign at follow up. Higher rates of recall were reported by were reported by younger and more tertiary educated women (Fig. 1). Adjusted analyses using logistic models were similar, with age remaining significant; compared to younger adults, middle-aged adults were less likely to recall the TGC campaign (AdjOR 0.60, 95%CI 0.41–0.89), as were older adults (AdjOR 0.51, 95% CI 0.35–0.74). Level of education was borderline significant (p = 0.051); with the tertiary educated more likely to recall the campaign, (AdjOR 1.36, 95%CI 0.99–1.86). Other demographic characteristics including income, country of birth, rural residence, Indigenous status, having children and reported disability were not associated with awareness of TGC-V.

Finally, the factors associated with any changes in physical activity days and the perception of feeling judged whilst playing sport are shown in Table 3. There were 31.7% of the low-active women who reported an increase in physical activity by ≥ 1 day (mean change 0.19 days (1.68)), and 38% who reported any decrease in feeling judged on the single item question (mean change − 0.25 (2.52)). Factors associated with those who made a positive change are shown in Table 3. Older women were less likely to make any change on either physical activity or being judged, but no other demographic characteristics were significant. Recalling the TGC-V message or logo was not related to change in physical activity or feeling judged. Additional analyses (Supplementary Table 3) show these analyses for women who made much larger changes in physical activity or decreases in feeling judged, decreases in the ‘barriers-feeling judged’ or ‘worry-feeling judged’ scores, and results were very similar, with no significant associations with TGC-V campaign recall.

## Discussion

Lower rates of physical activity among women are likely to be influenced by attitudes and perceptions of being judged, and by a lack of confidence in being active. These factors may be amenable to influence by a mass media campaign, and hence these results are important in long-term public health approaches to reducing inequalities in physical activity by gender. The initial wave of the VicHealth “This Girl Can” campaign showed some promising effects on perceptions of being judged, with reductions in feeling embarrassed. No substantial changes were noted in other attitudes, beliefs or self-efficacy, with limited changes in physical activity measures or in stages of change. TGC-V was recognised by around a third of women in this first post campaign assessment, prompted recall that is typical of physical activity campaigns[32]. Younger women, targeted in this campaign, were more likely to recall the specific campaign message or theme. As this was only the first wave of a multi-year TGC-V campaign, subsequent improvements in recognition across serial iterations of the campaign might be expected [33]. Nonetheless, the overall effects of wave one of TGC-V were modest, with similar effects seen in the cohort and independent sample data. However, although effects in the independent samples were in similar directions, they were smaller than in the cohort, indicating the possibility of a learning or pre-test sensitisation effect in the cohort due to repeat administration of the questions.

The rationale for both the UK TGC and the TGC-V campaigns was the consistent gender gap in sport and exercise across populations and over time [34, 35] and that this may be moderated by anxiety symptoms [36]. This inequality can potentially be influenced by mass communications campaigns that target gender stereotypes by showcasing women with a variety of body shapes and sizes being physically active, and the different ways people can engage in physical activity, to shift norms and reduce barriers to being active amongst women.

Understanding women’s experience of physical activity has been under-researched. There have been formative research studies that have examined the impact of these messages in experimental small samples. One such study showed that 339 women exposed to TGC depictions of exercise showed improved body satisfaction and physical activity intention compared to controls [37]. Population level barriers to women remain as one possible reason for gender differentials in physical activity; and exploring this was central to the formative research underpinning the TGC-V and the Sport England TGC campaigns [9–11].

The TGC-V campaign followed on from the extensive efforts of the Sport England “This Girl Can” campaign. This effort in England was a huge multi-strategy initiative alongside an extensive mass media campaign, aiming to change “how women think and feel about exercise and sport… And the fear of being judged” [10, 11]. No results have been reported in the peer-reviewed literature from this campaign, but Sport England reports suggest high rates of social media visits, high campaign awareness and up to 1.6 million additional women reporting that they initiated some exercise [10, 33]. This latter finding was corroborated by the independent representative Active People surveys, which showed decreases in the gender gap in physical activity coincident with the initial waves of the UK “This Girl Can” campaign [38]. The large increase in media reporting of TGC (UK) were followed by an increase in media reporting in Australia following TGC-V (Supplementary material, figure S1).

In Australia, many campaigns have addressed physical inactivity since the 1970s [39], but few have focused specifically on barriers among women. One recent initiative was the national “Girls Make Your Move” campaign between 2016 and 2018. This campaign targeted adolescent girls aged 12 to 20 years, and results have only been reported in government documents [40], but they did show a reported increase in understanding of information sources for where to be active, and feelings of being judged decreased from 28 to 17%, with no other changes in other attitudes or statements [40].

The TGC-V was the first reported adaptation and replication of the UK TGC messages in a mass-reach campaign. VicHealth initiated TGC-V in 2018, with a multi-year commitment to deliver a communitywide campaign across the state of Victoria. The TGC-V mass media component was strengthened through partnering with sport organisations and the Victorian Department of Sport and Recreation, through the use of community champions and role-models, and by linking to local community physical activity and sporting events.

The strengths of this evaluation are a large sample, with substantial diversity by sociodemographic attributes among the women surveyed. However, the sample was derived from a market research panel, but closely resembled all Victorian women. Psychological and attitude measures used were established and previously validated, although minor adaptations were made to the Theory of Planned Behaviour and Self-Determination Theory scales. Nonetheless, these measures showed good internal consistency. The self-report physical activity measure used here has been validated [26]. A further strength was the inclusion of both independent samples and cohort analyses, and the inclusion of two pre-campaign measures. The study used established and internally consistent measures of attitudes and beliefs and routine measures of campaign recall and awareness. The weaknesses are the modest effect observed, and the lack of a comparison region, although two baselines does provide some internal comparator. Whilst encouraging, these effects need to be monitored over time in subsequent campaign years to assess if further changes in the sense of being judged occurred and whether population changes in physical activity and sport occur among women.

## Conclusion

Large scale mass-reach campaigns are an expensive public health investment and need to be carefully evaluated. Previous TGC campaigns in England have only provided government reports of their effects, but they attracted much community interest and support. The VicHealth licencing and adaptation for Australian women was supported by a robust evaluation design and indicated initial positive effects on campaign awareness of the initiative and in a reduction in feelings of being judged by these low-active Victorian women. This provides initial evidence for the use of mass media campaigns to influence community perceptions among women, as a precursor to population behaviour change.

**Table 1:** Baseline demographic data at the pre-campaign measurement (%).

Demographic data	Whole cohort sample (n = 1032)	Key campaign Target group: ‘low active’ subset of cohort (n = 818)*	Independent samples (n = 2861) ¶
Age category 18–34 years (%) 35–49 years (%) 50 + years (%)	25.1 32.5 42.4	24.9 32.6 42.4	49.9 30.2 19.9
Partner /single (% with partner)	62.1	63.2	62.3
Any children in household (%)	46.1	47.1	47.7
Education (% tertiary education)	38.9	38.5	36.1
Occupation (% employed)	55.9	54.3	53.2
Income (% <$80,000 p.a.)	43.6	41.8	44.0
Country of birth (Australia, %)	80.7	80.4	78.0
Aboriginal or Torres Strait Islander (%)	1.1	1.1	3.1
Reported disability that limits movement (%)	25.6	27.5	25.4
Location (% metropolitan)	76.4	78.5	73.1
Low active: 0–4 days/week (%) Active: 5 + days/week 30 min (%)	79.3 20.7	(100)	80.5 19.5

Legend: * primary sample used in this analysis is the cohort sample who are low-active at baseline 1, those reporting < 5 days of activity in the past week

¶ independent samples comprised 976 women only surveyed at the first baseline, 904 surveyed only at the second baseline, and 981 different women surveyed at the post campaign survey; results for the independent samples are reported in Supplementary online material.

**Table 2:** Attitudes, barriers and behaviour change scores before and after the TGC-V campaign in the low-active cohort (n = 818)

Scores	Analysis confined to those low/inactive at baseline (n = 818)		Effect size (d) Ψ
**Physical activity measures**		Means (sd)	
Number of reported days/ week of 30 min (mean, sd) (physical activity single item) ^∑^	Baseline B1 Baseline B2 Post campaign	*1.67 (1.38)* 2.05 (1.79)** 1.86 (1.77)**	*0.24* *0.12*
Reported any exercise in the past week (%)		*B1% B2% Post % 54.8 55.6 56.1*	
Stage of change measure	Precontemplation Contemplation Preparation Action/maintenance	*B1% B2% Post%* *19.2 18.5 22.3* *17.5 14.9 16.4* *9.4 10.2 8.9* *53.9 56.4 52.3*	
**Measures of feeling judged**			
Feel judged as a barrier to sport and exercise participation (3 items) (lower score = less judged)	Baseline B1 Baseline B2 Post campaign	*13.86 (8.06)* 13.43 (7.89)** 12.66 (8.14) **	*0.05* *0.15*
Feeling judged causing worry: 3 items, increased score is more worried	Baseline B1 Baseline B2 Post campaign	*9.95 (7.78)* *9.81 (7.61)* *9.52 (7.63)*	*0.02* *0.06*
Single item ‘how worried about feeling judged in exercise/sport’: rated 1–10, increased score more judged	Baseline B1 Baseline B2 Post campaign	*4.26 (3.08)* *4.11 (3.09)* 4.01 (3.00)**	*0.05* *0.08*
**Other psychosocial measures**			
Exercise relevance score 7 items; higher score is increased relevance	Baseline B1 Baseline B2 Post campaign	**Mean (sd)** 49.61 (10.89) 49.46 (12.01) 50.08 (11.03)	0.01 0.04
Theory planned behaviour score: 6 items, higher score is increased positive attitude to exercise	Baseline B1 Baseline B2 Post campaign	37.92 (13.77) 38.06 (14.29) 38.58 (13.88)	0.001 0.05
Self-determination theory score: 4 items, higher score is more self determination	Baseline B1 Baseline B2 Post campaign	13.49 (3.82) 13.58 (3.92) 13.73 (3.80)*	0.02 0.06
Physical activity efficacy score: 3 items, higher score is greater situation specific confidence	Baseline B1 Baseline B2 Post campaign	16.51 (7.77) 16.30 (8.03) 16.22 (7.99)	0.03 0.05
Embarrassed score: 7 items ( decreased score is less embarrassment)	Baseline B1 Baseline B2 Post campaign	30.91 (16.18) 29.45 (16.32) 28.97 (16.32) *	0.09 0.12

**Legend**: B1 first baseline, B2 second baseline, post campaign

Univariate GLM, Tukey post-hoc contrasts: compared to baseline B1 *p < 0.05 ** p < 0.01

Ψ Effect sizes (Cohen’s d), comparing B1/B2; B1/post.

∑: using nonparametric methods for PA days: B1-B2 KW-H statistic 1.58, p = 0.20; B1-post KW-H = 5.22, p = 0.07; B2-post KW-H = 5.13, p = 0.02.

**Table 3:** Factors associated with any change in physical activity days or feeling judged from baseline 1 to post campaign among low-active women (n = 818)

Variable	Change in PA days	Change in single item ‘feeling judged during exercise/sport’
Mean change score (SD)	+ 0.19 days (1.68)	-0.25 (2.52)
Any positive change	increased by ≥ 1 day (31.7%)	Reduced score by ≥ 1 unit (38.0%)
Correlates of positive change	Adj ORs* (95%CI)	Adj ORs (95%CI)
Age 18–29 30–49 50+	1.0 0.89 (0.61–1.33) **0.57 (0.39–0.84)**	1.0 0.75 (0.51–1.10) **0.59 (0.40–0.84)**
Education: school ≥ Tertiary	1.0 1.19 (0.87–1.63)	1.0 0.95 (0.70–1.29)
Children No Yes	1.0 0.87 (0.64–1.18)	1.0 0.99 (0.74–1.34)
Recall TCG-V No Yes	1.0 0.98 (0.72–1.36)	1.0 0.83 (0.60–1.18)

*Adjusted odds ratios, adjusted for all other variables

## Electronic supplementary material

Below is the link to the electronic supplementary material.

Supplementary Material 1

## Data Availability

The data generated and analysed during the current study are not publicly available due to being owned by VicHealth (Government of Victoria), but are available on reasonable request from the corresponding author. Materials relevant to this campaign are available from the VicHealth website: https://thisgirlcan.com.au/.

## Declarations

All methods were carried out in accordance with relevant guidelines and regulations.
