# Supplementary material for: Impact of the first year of the “This girl can” physical activity and sport mass media campaign in Australia
Source: BMC Public Health. 2023 Feb 15;23:333. doi: 10.1186/s12889-023-15091-2 (PMC9930268; doi:10.1186/s12889-023-15091-2)
Supplement: Supplementary file 1 — Supplementary Material 1 [file 12889_2023_15091_MOESM1_ESM.docx]

**Supplementary Appendix material**

**Supplementary material S1**

**Attitude and behaviour questions asked in This Girl Can (TGC-V) surveys**

***PHYSICAL ACTIVITY (PA) QUESTIONS***

**Single item PA question**

*In the past week, on how many days have you done a total of 30 minutes or more of physical activity, which was enough to raise your breathing rate? This may include sport, exercise and brisk walking or cycling for recreation or to get to and from places, but should not include housework or physical activity that may be part of your job.*

*0 days / 1 day / 2 days / 3 days / 4 days / 5days / 6 days/ 7 days*

When was the last time you exercised or played sport?

In the past week / last month / last 6 months / last 12 months / more than a year

***FEELING JUDGED ABOUT ACTIVITY/EXERCISE QUESTIONS***

**Barriers to exercise – perception of being judged**

There are many things that can get in the way of physical activity. Please rate how sure you are that you can exercise and/or play sport in each situation below.

|  | I’m sure I can’t  I’m sure I can | | | | |  | I’m sure I can | | | | |
| --- | --- | --- | --- | --- | --- | --- | --- | --- | --- | --- | --- |
| Exercise or play sport when I feel judged about my appearance | 0 | 1 | 2 | 3 | 4 | 5 | 6 | 7 | 8 | 9 | 10 |
| Exercise or play sport when I feel judged about having to stop or slow down | 0 | 1 | 2 | 3 | 4 | 5 | 6 | 7 | 8 | 9 | 10 |
| Exercise or play sport when I feel judged for making it a priority | 0 | 1 | 2 | 3 | 4 | 5 | 6 | 7 | 8 | 9 | 10 |

**Worry about being judged whilst exercising**

How worried are you about the following in relation to exercise and/or playing sport? Please respond on a scale of 0 to 10, where 0 equals ‘not at all worried’ and 10 equals ‘extremely worried’.

|  | Not at all worried  I’m sure I can | | | | |  | Extremely worried | | | | |
| --- | --- | --- | --- | --- | --- | --- | --- | --- | --- | --- | --- |
| Being judged by men | 0 | 1 | 2 | 3 | 4 | 5 | 6 | 7 | 8 | 9 | 10 |
| Being judged by other women | 0 | 1 | 2 | 3 | 4 | 5 | 6 | 7 | 8 | 9 | 10 |
| That people will judge me for prioritizing exercise over other things | 0 | 1 | 2 | 3 | 4 | 5 | 6 | 7 | 8 | 9 | 10 |

**Single item feeling judged**

How worried are you about feeling judged when exercising and/or playing sport? Please respond on a scale of 0 to 10, where 0 equals ‘not at all worried’ and 10 equals ‘extremely worried’.

| Not at all worried | | | |  | | | | | Extremely worried | |
| --- | --- | --- | --- | --- | --- | --- | --- | --- | --- | --- |
| 0 | 1 | 2 | 3 | 4 | 5 | 6 | 7 | 8 | 9 | 10 |

**Embarassed about exercise**

How worried are you about the following in relation to exercise and/or playing sport? Respond on a scale of 0 to 10, 0 equals ‘not at all worried’ and 10 equals ‘extremely worried’.

|  | **Not at all worried**  **I’m sure I can** | | | | |  | **Extremely worried** | | | | |
| --- | --- | --- | --- | --- | --- | --- | --- | --- | --- | --- | --- |
| Being sweaty | 0 | 1 | 2 | 3 | 4 | 5 | 6 | 7 | 8 | 9 | 10 |
| Wearing tight clothing | 0 | 1 | 2 | 3 | 4 | 5 | 6 | 7 | 8 | 9 | 10 |
| Showing my body | 0 | 1 | 2 | 3 | 4 | 5 | 6 | 7 | 8 | 9 | 10 |
| Not being feminine | 0 | 1 | 2 | 3 | 4 | 5 | 6 | 7 | 8 | 9 | 10 |
| Not being fit enough | 0 | 1 | 2 | 3 | 4 | 5 | 6 | 7 | 8 | 9 | 10 |
| Not being good enough | 0 | 1 | 2 | 3 | 4 | 5 | 6 | 7 | 8 | 9 | 10 |
| Beginning again after some time off | 0 | 1 | 2 | 3 | 4 | 5 | 6 | 7 | 8 | 9 | 10 |

***OTHER ATTITUDE AND BELIEF QUESTIONS***

**Stage of change**

Do you exercise and/or play sport at least at a moderate intensity for at least 30 minutes on 2 or more days per week (e.g. a brisk walk, recreational swimming, dancing, social tennis or golf, jogging, aerobics or fast cycling)?

| Yes, I do | 1 |
| --- | --- |
| No, I do not | 2 |

Which of the following best applies to you?

| I have been at this rate (at least 30 minutes on 2 or more days per week) or higher, but for **less** **than 6 months** | 1 |
| --- | --- |
| I have been at this rate (at least 30 minutes on 2 or more days per week) or higher for **6 months or more** | 2 |

Which of the following best applies to you?

| I do **not** intend to begin exercising or playing sport at this rate (at least 30 minutes on 2 or more days per week) in the **next 6 months** | 1 |
| --- | --- |
| I **do** intend to begin exercising or playing sport at this rate (at least 30 minutes on 2 or more days per week) in the **next 6 months** | 2 |
| I **do** intend to begin exercising or playing sport at this rate (at least 30 minutes on 2 or more days per week) in the **next 30 days** | 3 |

**Physical activity Self efficacy**

How confident are you about being able to complete **30 minutes of exercise or sport on at least 2 days per week** at least at moderate intensity (e.g. a brisk walk, recreational swimming, dancing, social tennis or golf, jogging, aerobics or fast cycling)?

| **Not at all confident** | | | |  | | | | | **Extremely confident** | |
| --- | --- | --- | --- | --- | --- | --- | --- | --- | --- | --- |
| 0 | 1 | 2 | 3 | 4 | 5 | 6 | 7 | 8 | 9 | 10 |

How confident are you about being able to complete at least **150 minutes of physical activity per week** at least at moderate intensity (e.g. a brisk walk, recreational swimming, dancing, social tennis or golf, jogging, aerobics or fast cycling)?

| **Not at all confident** | | | |  | | | | | **Extremely confident** | |
| --- | --- | --- | --- | --- | --- | --- | --- | --- | --- | --- |
| 0 | 1 | 2 | 3 | 4 | 5 | 6 | 7 | 8 | 9 | 10 |

How confident are you about trying new forms of exercise and/or sport?

| **Not at all confident** | | | |  | | | | | **Extremely confident** | |
| --- | --- | --- | --- | --- | --- | --- | --- | --- | --- | --- |
| 0 | 1 | 2 | 3 | 4 | 5 | 6 | 7 | 8 | 9 | 10 |

**Exercise relevance**

The following statements are things some people have said about exercise and/or playing sport. How much do you agree or disagree with each one?

|  | **Strongly disagree** | | | | | **Neither Agree nor Disagree** | **Strongly agree** | | | | |
| --- | --- | --- | --- | --- | --- | --- | --- | --- | --- | --- | --- |
| I feel that exercise is for me | 0 | 1 | 2 | 3 | 4 | 5 | 6 | 7 | 8 | 9 | 10 |
| I feel that playing sport is for me | 0 | 1 | 2 | 3 | 4 | 5 | 6 | 7 | 8 | 9 | 10 |
| It is important for women to be physically active | 0 | 1 | 2 | 3 | 4 | 5 | 6 | 7 | 8 | 9 | 10 |
| Being physically active is good for your mental health | 0 | 1 | 2 | 3 | 4 | 5 | 6 | 7 | 8 | 9 | 10 |
| Exercise and sport are for women as much as for men | 0 | 1 | 2 | 3 | 4 | 5 | 6 | 7 | 8 | 9 | 10 |
| You don’t have to be fit to begin exercise or sport | 0 | 1 | 2 | 3 | 4 | 5 | 6 | 7 | 8 | 9 | 10 |
| I feel like working up a sweat during exercise and/or playing sport is something that is more appropriate for males than females | 0 | 1 | 2 | 3 | 4 | 5 | 6 | 7 | 8 | 9 | 10 |
| I live in an area where there are many options for women to engage in exercise and/or play sport | 0 | 1 | 2 | 3 | 4 | 5 | 6 | 7 | 8 | 9 | 10 |

**Theory of planned behaviour**

Please indicate how you feel about the following as they relate to exercise and/or playing sport.

|  | **Strongly disagree** | | | | | **Neither Agree nor Disagree** | **Strongly agree** | | | | |
| --- | --- | --- | --- | --- | --- | --- | --- | --- | --- | --- | --- |
| I feel positively toward exercise and/or playing sport | 0 | 1 | 2 | 3 | 4 | 5 | 6 | 7 | 8 | 9 | 10 |
| People I am close to feel positively toward exercise and/or playing sport | 0 | 1 | 2 | 3 | 4 | 5 | 6 | 7 | 8 | 9 | 10 |
| People I am close to feel positively about me exercising and/or playing sport | 0 | 1 | 2 | 3 | 4 | 5 | 6 | 7 | 8 | 9 | 10 |
| I could be successful at exercise and/or playing sport | 0 | 1 | 2 | 3 | 4 | 5 | 6 | 7 | 8 | 9 | 10 |
| I intend to participate in exercise and/or sport in the next 12 months | 0 | 1 | 2 | 3 | 4 | 5 | 6 | 7 | 8 | 9 | 10 |
| My friends and/or family support me to exercise and/or play sport | 0 | 1 | 2 | 3 | 4 | 5 | 6 | 7 | 8 | 9 | 10 |

**Self determination theory**

Please think about your participation in exercise and/or sport, and indicate your feeling from ‘not at all’ to ‘very much’, as they relate to the following four statements: When I exercise or play sport I feel …

|  | **Not at all** |  |  |  | **Very much** |
| --- | --- | --- | --- | --- | --- |
| That my choices are based on my personal interests and values | 1 | 2 | 3 | 4 | 5 |
| Very capable in what I do | 1 | 2 | 3 | 4 | 5 |
| A strong sense of connection with the people I spend time with while exercising or playing sport | 1 | 2 | 3 | 4 | 5 |
| Quite satisfied with who I am while exercising or playing sport | 1 | 2 | 3 | 4 | 5 |

**Table S2. Whole cohort sample and independent samples: attitudes, barriers and behaviour change scores before and after the TGC-V campaign (N=1032)**

| **Score** | **Whole cohort sample (n=1032)**  (low-active and sufficiently active) | | **Independent samples, low-active women (N=2861)** | | |
| --- | --- | --- | --- | --- | --- |
| **Physical activity measures** | | | | | |
| Physical activity days/week | Baseline B1  Baseline B2  Post campaign | Mean (sd)  2.54 (2.14)  2.53 (2.09)  2.42 (2.13)* | Baseline B1 (n=976)  Baseline B2 (n=904)  Post campaign (n=981) | | Mean (sd)  **2.66 [2.08]**  **2.77 [2.09]**  **2.51 [2.11]** |
| Report any exercise in past week (%) | Proportion, 95%CI  %B1 %B2 %post  61.9 61.3 61.9 | | Proportion, 95%CI  %B1 %B2 %post  55.7 59.4 55.0 | | |
| Stage of change^  Action/maintenance | %B1 %B2 %post  62.5 63.3 59.7 | | % action and maintenance  %B1 %B2 %post  61.2 64.6 63.4 | | |
| **Measures of feeling judged** (lower scores less perception of being judged) | | | | | |
| Feel judged as a barrier to sport participation  3 items; (internal consistency α=0.91) | Baseline B1  Baseline B2  Post campaign | **Mean (sd)**  12.88 (8.15)  12.46 ((8.02)  11.97 (8.19)Ж | Baseline B1  Baseline B2  Post campaign | **Mean (sd)**  13.56 (8.20)  13.28 (8.21)  12.65 (7.73) * | |
| Feeling judged causing worry Ж: 3 items, (internal consistency α=0.79) | Baseline B1  Baseline B2  Post campaign | 9.66 (7.79)  9.42 (7.65)  9.09 (7.74)** | Baseline B1  Baseline B2  Post campaign | 10.22 (7.94]  9.35 [7.47]*  9.67 [7.46] | |
| Single item ‘how worried about feeling judged in exercise/sport’: self-rated 1-10, | Baseline B1  Baseline B2  Post campaign | 4.08 (3.09)  3.92 (3.11)  3.77 (3.05)* | Baseline B1  Baseline B2  Post campaign | 4.19 [3.26]  4.18 [3.14]  4.04 [3.10] | |
| **Other beliefs and attitudes** | | | | | |
| Exercise relevance score  7 items; higher score is increased relevance  (internal consistency α=0.78) | Baseline B1  Baseline B2  Post campaign | **Mean (sd)**  50.75 (10.9)  50.63 (11.9)  51.25 (11.2) | Baseline B1  Baseline B2  Post campaign | **Mean** **(sd)**  50.67 [13.52]  51.87 [12.30]  51.19 [12.32] | |
| Theory planned behaviour score: 6 items, higher score is increased positive attitude to exercise (internal consistency α=0.92) | Baseline B1  Baseline B2  Post campaign | 39.47 (13.78)  39.58 (14.03)  40.17 (13.81)* | Baseline B1  Baseline B2  Post campaign | 40.63 [14.00]  42.35 [13.19]  41.25 [13.12] | |
| Self-determination theory score: 4 items, higher score is more self-determination (internal consistency α=0.84) | Baseline B1  Baseline B2  Post campaign | 13.94 (3.84)  14.06 (3.86)  14.14 (3.79)* | Baseline B1  Baseline B2  Post campaign | 14.50 [3.72]  14.71 [3.64]  14.46 [3.67] | |
| Efficacy score: 3 items, higher score is greater situation specific confidence (internal consistency α=0.85) | Baseline B1  Baseline B2  Post campaign | 17.91 (7.84)  17.54 (8.05)  17.46 (8.02)^ | Baseline B1  Baseline B2  Post campaign | 17.61 [8.52]  18.26 [7.97]  127.77 [7.93] | |
| Embarrassed score ¶: 7 items (decreased score is less embarrassment) (internal consistency α=0.88) | Baseline B1  Baseline B2  Post campaign | 29.96 (16.67)  28.37 16.58)**  27.41 16.72)** | Baseline B1  Baseline B2  Post campaign | 29.40 [16.57]  28.52 [16.54]  28.80 [16.48] | |

**Legend** *p<0.05 **p<0.01 ^p<0.05 (worsened score)

¶ embarrassed score, F=6.20, post hoc Tukey contrasts, B1/post p<0.01; Cohens d=0.15; Ж feeling judged F=3.2, contrasts B1/post p=0.03 ; Cohens d=0.11.

(in addition) Campaign Recall in the independent samples: campaign recall was 12.6% (B1), 12.2% (B2) and 32.0% at the three time points, very similar to the cohort sample.

**Supplementary Material Figure S1. Factiva* (media monitoring database): the number of media reports of (“This Girl Can” and “campaign”) over time in the UK/England and Australia/Victoria/Melbourne**

*Factiva database, <https://www.dowjones.com/professional/factiva/> accessed through the University of Sydney library; Factiva is an established database for reports on mainstream newspapers, wire service reports, websites.

**Supplementary Table 3: Factors associated with substantial changes in physical activity or the single item ‘feeling judged’, or associated with the other ‘feeling judged’ measures (n=818)**

|  | **Variables used in Table 3 but much greater changes** | | **Substantial change in other ‘feeling judged’ measures** | |
| --- | --- | --- | --- | --- |
| Variable | Any change in PA and meeting PA guideline of 5 days/week | Substantial change in *Single item ‘feeling judged* in exercise/sport’ | Substantial change in *Feel judged as a barrier* influenced sport participation | Substantial change in *Feeling judged that caused worry* |
| Mean change score (SD) | +0.19 days (1.68) | -0.25 (2.52) | -1.20 (6.98) | -0.43 (6.25) |
| Substantial change (% of sample) | ≥ 1 day ⇧ in PA and met PA guideline (8.4% ) | ≥ 3.00 units ⇩ (14.9%) | ≥ 6.00 units ⇩ (17.4%) | ≥ 6.00 units ⇩ (17.6%) |
| Characteristics | Adj ORs (95%CI) | Adj ORs (95%CI) | Adj ORs (95%CI) | Adj ORs (95%CI) |
| Age 18-29  30-49  50+ | 1.0  1.15 (.60 – 2.02)  0.87 (.45-1.67) | 1.0  1.06 (.63-1.76)  0.76 (.46-1.37) | 1.0  1.44 (.86-2.41)  1.27 (.77-2.08) | 1.0  1.01 (.63-1.61)  0.70 (44-1.13) |
| Education: school  ≥ Tertiary | 1.0  1.35 (.81-2.26) | 1.0  0.95 (.63-1.42) | 1.0  0.84 (.56-1.25) | 1.0  1.04 (.71-1.52) |
| Children No  Yes | 1.0  0.91 (.54-1.52) | 1.0  1.02 (.69-1.53) | 1.0  0.98 (.68-1.43) | 1.0  1.31 (.90-1.90) |
| Recall TCG-V No  Yes | 1.0  1.03 (.60-1.75) | 1.0  0.81 (.52-1.25) | 1.0  0.71 (.47-1.07) | 1.0  0.87 (.58-1.29) |
